# Biomechanical Effects of tPRK, FS-LASIK, and SMILE on the Cornea

**DOI:** 10.3389/fbioe.2022.834270

**Published:** 2022-03-31

**Authors:** Yue Xin, Bernardo T. Lopes, JunJie Wang, Jie Wu, ManMan Zhu, MuChen Jiang, YuanYuan Miao, HuiNi Lin, Si Cao, XiaoBo Zheng, Ashkan Eliasy, ShiHao Chen, QinMei Wang, YuFeng Ye, FangJun Bao, Ahmed Elsheikh

**Affiliations:** ^1^ Eye Hospital, Wenzhou Medical University, Wenzhou, China; ^2^ School of Engineering, University of Liverpool, Liverpool, United Kingdom; ^3^ The Institute of Ocular Biomechanics, Wenzhou Medical University, Wenzhou, China; ^4^ STU-CUHK Joint Shantou International Eye Center, Shantou, China; ^5^ Wuhan Puren Hospital, Wuhan, China; ^6^ National Institute for Health Research (NIHR) Biomedical Research Centre for Ophthalmology, Moorfields Eye Hospital NHS Foundation Trust and UCL Institute of Ophthalmology, London, United Kingdom; ^7^ Beijing Advanced Innovation Center for Biomedical Engineering, Beihang University, Beijing, China

**Keywords:** biomechanical response, *in-vivo*, tPRK, FS-LASIK, SMILE

## Abstract

**Purpose:** The objective of this study is to evaluate the *in vivo* corneal biomechanical response to three laser refractive surgeries.

**Methods:** Two hundred and twenty-seven patients who submitted to transepithelial photorefractive keratectomy (tPRK), femtosecond laser-assisted *in-situ* keratomileusis (FS-LASIK), or small-incision lenticule extraction (SMILE) were included in this study. All cases were examined with the Corvis ST preoperatively (up to 3 months) and postoperatively at 1, 3, and 6 months, and the differences in the main device parameters were assessed. The three groups were matched in age, gender ratio, corneal thickness, refractive error corrections, optical zone diameter, and intraocular pressure. They were also matched in the preoperative biomechanical metrics provided by the Corvis ST including stiffness parameter at first applanation (SP-A1), integrated inverse radius (IIR), deformation amplitude (DA), and deformation amplitude 2 mm away from apex and the apical deformation (DARatio2mm).

**Results:** The results demonstrated a significant decrease post-operation in SP-A1 and significant increases in IIR, DA, and DARatio2mm (*p* < 0.05), all of which indicated reductions in overall corneal stiffness. Inter-procedure comparisons provided evidence that the smallest overall stiffness reduction was in the tPRK group, followed by the SMILE, and then the FS-LASIK group (*p* < 0.05). These results remained valid after correction for the change in CCT between pre and 6 months post-operation and for the percentage tissue altered. In all three surgery groups, higher degrees of refractive correction resulted in larger overall stiffness losses based on most of the biomechanical metrics.

**Conclusion:** The corneal biomechanical response to the three surgery procedures varied significantly. With similar corneal thickness loss, the reductions in overall corneal stiffness were the highest in FS-LASIK and the lowest in tPRK.

## Introduction

The current growth in interest in the evaluation of corneal biomechanics and the changes caused by laser vision correction (LVC) surgeries is driven mainly by the emergence of cases that developed iatrogenic ectasia (Randleman, 2016). This complication is multifactorial, and some of its risk factors are still to be elucidated (Binder, 2007; Bohac et al., 2018). The corneal instability leading to irregular astigmatism and vision impairment in this complication can be triggered in susceptible corneas by the tissue alterations and the subsequent stiffness reductions associated with the surgical procedures (Ambrósio et al., 2010). Bohac et al. (2018) found that corneal thickness (a major contributing factor to cornea’s overall stiffness) below 500 µm was present in 50% of the ectatic cases. Other researchers further noted that corneal instability can develop in previously stiff and stable corneas when the tissue alteration caused by the procedure was large (Santhiago et al., 2014; Santhiago et al., 2015). In connection with this observation, Bohac et al. (2018) identified risk factors related to the tissue alteration induced by procedures such as a low residual stromal bed (≤300 µm) in 30% of the cases and high percentage tissue thickness alteration (≥40%) in 20% (Bohac et al., 2018). Interestingly, these studies have shown that the majority of LASIK cases with these risk factors remained stable for a period between 2 and 8 years before developing ectasia, suggesting that still unknown factors affecting the corneal biomechanical behavior were involved in this complication.

Over the years, and with more cases of iatrogenic ectasia reported (Ambrósio, 2019), there was a search for procedures that would have a low impact on corneal biomechanics—this search led to an increased use of surface ablation surgeries including transepithelial photorefractive keratectomy (tPRK) and the introduction of the small-incision lenticule extraction (SMILE) surgery. However, recent case reports of iatrogenic ectasia post-SMILE have highlighted the need to carefully evaluate the biomechanical advantage of this procedure over laser-assisted *in-situ* keratomileusis (LASIK) (El-Naggar, 2015; Wang et al., 2015; Mattila and Holopainen, 2016; Voulgari et al., 2018; Pazo et al., 2019; Shetty et al., 2019). Even though there is experimental and numerical evidence of the reduced biomechanical effect of SMILE compared to LASIK (Seven et al., 2017; Spiru et al., 2018), this expected reduced effect was not evident in clinical studies (Guo et al., 2019; Raevdal et al., 2019). The same is true for comparisons between LASIK and SMILE on one hand and PRK on the other. Although PRK did not involve tissue separation, or the formation of a cap or a flap, its expected reduced biomechanical effect compared to both LASIK and SMILE was not consistently seen in earlier clinical studies (Chen et al., 2016; Yildirim et al., 2016; Spiru et al., 2018; Yu et al., 2019).

With the fast growth in popularity of laser vision correction surgeries worldwide (Kim et al., 2019), the increased recognition of the effect of corneal biomechanics on surgery outcomes (Roberts, 2016; Esporcatte et al., 2020), and the continued concern about post-surgery ectasia, it is important to reach a definitive answer concerning the mechanical effect of different LVCs. This study is part of our efforts to evaluate clinically the *in vivo* biomechanical impact of the three most common forms of LVCs, namely, femtosecond LASIK (FS-LASIK), SMILE, and tPRK, while controlling for potential confounding factors.

## Materials and Methods

### Patient Inclusion

This prospective comparative case series was approved by the Ethics Committee of the Eye Hospital, Wenzhou Medical University (WMU). Two hundred and twenty-seven Chinese patients, who had undergone corneal refractive surgery for myopia and astigmatism at the Eye Hospital of WMU, were included in this study. All the patients belong to east Asian race and the Han nationality. The patients had myopia between −1.00 and −9.75 D (mean −4.82 ± 1.57 D) and astigmatism between 0 and −3.00 D (mean −0.76 ± 0.59 D). Among these patients, 74 underwent tPRK, 81 accepted FS-LASIK, and 72 underwent SMILE. Informed consent was provided by all participants to use their data in research. Only one eye, randomly selected per patient, was included in the analysis. All the LVC was operated by the same operator, and only patients with no systemic or ocular condition apart from the refractive error and with complete records of the surgical procedure, clinical examination, and Corvis ST (CVS, software version 1.6r2031, OCULUS Optikgeräte, Wetzlar, Germany) results up to 3 months on preoperative (pre), 1 month (pos1m), 3 months (pos3m) and 6 months postoperative (pos6m) were included. Those who did not complete the 6 months postoperative follow-up were excluded from the study.

### Surgical Technique

In the tPRK procedure, the epithelium and stroma were ablated with a central ablated epithelium thickness of 55 μm in a single step using the aberration-free mode of the Schwind Amaris 750 Hz excimer laser (Schwind eye-tech-solutions, Kleinostheim, Germany). In the FS-LASIK procedure, the lamellar flap was created with a femtosecond laser (Ziemer Ophthalmic Systems AG, Port, Switzerland). The flaps had a superior hinge, and their thickness ranged from 95 to 110 µm and diameter from 8.5 to 9.0 mm. The FS-LASIK ablation was then performed using the Schwind Amaris 750 Hz excimer laser. The SMILE procedure was performed using the VisuMax femtosecond laser (Carl Zeiss Meditec, Jena, Germany). A stromal lenticule was removed, leaving a cap whose thickness ranged from 115 to 140 µm.

The postoperative care was similar for the three procedures starting with one drop of tobramycin/dexamethasone (Tobradex; Alcon, TX, United States) instilled at the surgical site. This was followed by placing a bandage contact lens (Acuvue Oasys; Johnson & Johnson, FL, United States) on the cornea and keeping it for 1 day in FS-LASIK and for 3–7 days in the tPRK group until complete corneal re-epithelialization. Fluorometholone 0.1% (Flumetholon; Santen, Osaka, Japan) and topical levofloxacin 0.5% (Cravit; Santen, Osaka, Japan) were then applied four times a day in all three groups. In the tPRK group, the fluorometholone dosage was tapered each subsequent 2–3 weeks and stopped 2–3 months after surgery, while for FS-LASIK and SMILE, the fluorometholone dosage was tapered each subsequent week until 1 month after surgery.

Surgical parameters including optical zone diameter (OZD) and manifest refractive error correction (MRx) were recorded from surgery planning/treatment printouts. The MRx was recorded with spherical (MRxSph) and cylindrical parts (MRxCyl) and was converted into spherical equivalent (SE). Central corneal thickness (CCT) and mean corneal curvature (Km) were measured with a Pentacam (software version: 1.21r65, OCULUS Optikgerate GmbH, Wetzlar, Germany) and used to calculate the difference in CCT (CCT_dif_) between the values obtained before and 6 months after surgery. The CCT measurements also allowed calculation of the tissue altered (TA) as TA = CCT_dif_ in the tPRK group, TA = flap thickness + CCT_dif_ in the FS-LASIK group, and TA = cap thickness + CCT_dif_ in the SMILE group. PTA was defined as percentage tissue altered (PTA) as PTA = TA/CCT_pre_. According to SE measured pre surgery, participants were divided into two groups with low-to-moderate myopia (−0.50 D > SE ≥ −5.00 D, LM group) and high myopia (−5.00 D > SE, HM group) as we did in a previous study (Bao et al., 2020). No patients experienced complications related to the surgical procedures.

### Biomechanical Evaluation

All Corvis ST exams were taken in the sitting position with undilated pupils by two experienced examiners. They were taken in the same half-day session to minimize diurnal effects. Five Corvis ST biomechanical metrics that had been linked to corneal stiffness (Vinciguerra et al., 2016; Roberts et al., 2017) were recorded pre- and post-surgery. The parameters included the stiffness parameter at first applanation (SP-A1) (Roberts et al., 2017), calculated as the difference between the adjusted air puff pressure at first applanation (AdjAP1) and biomechanically corrected intraocular pressure (bIOP) divided by the defection amplitude at the first applanation (A1DeflAmp).

SP-A1=(adjAP1-bIOP)/(A1DeflAmp) (1)

The metrics also included the integrated inverse radius (IIR)—the integrated sum of the inverse concave radii between the first and second applanation events; the deformation amplitude (DA), which measures the corneal apex maximum displacement under air-puff; and the ratio between the deformation amplitude 2 mm away from apex and the apical deformation (DARatio2mm). These parameters have been described as suitable parameters to evaluate corneal biomechanics *in vivo* (Vinciguerra et al., 2016).

### Statistical Analysis

All statistical analyses were performed using PASW Statistics 20.0 (SPSS Inc., Chicago, United States). Baseline characteristics among the three surgery groups were paired using propensity density scores in order to reduce the influence of confounding factor. Comparisons among the three surgery groups were made using the MANOVA of repeated measurements. One-way ANOVA and ANCOVA (analysis of covariates) with a general linear model were used to compare the changes in biomechanical metrics in the three surgery groups, where CCT_dif_ or PTA was considered a covariate. The frequencies of the categorical variable gender were arranged in a 3 × 2 contingency table, and the chi-square test of independence was used to compare them. A *p*-value of less than 0.05 was considered statistically significant.

## Results

The three groups (tPRK, FS-LASIK, and SMILE) were matched in age (27.2 ± 5.2 years vs. 26.1 ± 4.8 years and 26.3 ± 6.0 years, *F* = 0.876, *p* = 0.418), gender ratio (male/female: 22/52 vs. 37/44 and 33/39, *χ* ^2^ = 5.312, *p* = 0.070), CCT (540.1 ± 29.3 μm vs. 545.0 ± 27.2 μm and 546.6 ± 21.5 μm, *F* = 1.838, *p* = 0.162), MRxSph (−4.69 ± 1.57 D vs. −5.04 ± 1.58 D and −4.72 ± 1.57 D, *F* = 1.151, *p* = 0.318), MRxCyl (−0.81 ± 0.57 D vs. −0.66 ± 0.57 D and −0.83 ± 0.63 D, *F* = 1.776, *p* = 0.172), OZD (6.59 ± 0.37 mm vs. 6.61 ± 0.34 mm and 6.61 ± 0.18 mm, *F* = 0.133, *p* = 0.876), and bIOP (13.90 ± 1.83 mmHg vs. 14.17 ± 1.91 mmHg and 13.67 ± 1.68 mmHg, *F* = 1.456, *p* = 0.235). Record matching was also applied for the LM group and HM group individually except for CCT_dif_ (Table 1).

**TABLE 1:** Basic information for the three surgery groups.

Biometric parameter	Groups	tPRK	FS-LASIK	SMILE	*F*/*χ* (Binder, 2007)	*p*
Age	LM group	27.0 ± 5.0	26.4 ± 4.6	25.7 ± 6.3	0.558	0.574
	HM group	27.3 ± 5.4	25.8 ± 5.1	26.9 ± 5.7	0.850	0.430
Gender ratio	LM group	12/25	17/17	20/14	5.183	0.075
	HM group	10/27	20/27	13/25	2.213	0.331
CCT, µm	LM group	539.1 ± 33.4	541.6 ± 24.5	546.0 ± 20.3	1.687	0.191
	HM group	541.0 ± 25.5	547.6 ± 29.1	547.0 ± 22.7	0.381	0.684
MRxSph, D	LM group	−3.47 ± 0.84	−3.62 ± 0.87	−3.38 ± 0.72	0.757	0.472
	HM group	−5.91 ± 1.09	−6.06 ± 1.10	−5.93 ± 1.05	0.246	0.783
MRxCyl, D	LM group	−0.68 ± 0.49	−0.62 ± 0.64	−0.81 ± 0.77	0.802	0.451
	HM group	−0.94 ± 0.61	−0.70 ± 0.52	−0.84 ± 0.49	2.143	0.122
OZD, mm	LM group	6.77 ± 0.29	6.79 ± 0.25	6.68 ± 0.11	2.021	0.138
	HM group	6.42 ± 0.36	6.49 ± 0.34	6.56 ± 0.20	1.882	0.157
bIOP, mmHg	LM group	13.78 ± 1.93	13.68 ± 1.69	13.46 ± 1.93	0.273	0.762
	HM group	14.02 ± 1.75	14.52 ± 2.00	13.85 ± 1.42	0.148	0.863
CCT_dif_, µm	LM group	−75.0 ± 19.8	−71.0 ± 20.3	−74.2 ± 12.2	0.447	0.641
	HM group	−116.1 ± 25.6	−97.9 ± 22.8	−94.7 ± 18.7	9.787	<0.01**
Flap/cap* thickness, µm	LM group	—	102.5 ± 4.7	120.6 ± 3.4	333.5	<0.01**
	HM group	—	100.9 ± 2.8	119.7 ± 1.1	1504.9	<0.01**

CCT, central corneal thickness; MRxSph and MRxCyl, spherical and cylindrical manifest refractive error correction, respectively; OZD, optical zone diameter; CCT_dif_, the difference in CCT between the values obtained before and 6 months after surgery.

Km decreased at pos1m compared with the pre-surgery stage in all surgery groups (all *p* < 0.01). During follow-up, Km values at pos3m and pos6m were significantly different in the tPRK group (*p* = 0.007), although there was little change from pos1m to pos3m (*p* > 0.05). In contrast, in the FS-LASIK group, Km experienced significant fluctuations during follow-up (pos1m vs pos3m: *p* < 0.01; pos1m vs. pos6m: *p* < 0.01; pos3m vs. pos6m: *p* = 0.019). Km values at pos1m and pos6m were also significantly different in the SMILE group (*p* = 0.020), but the changes were not significant within shorter follow-up stages (*p* > 0.05).

**FIGURE 1:**
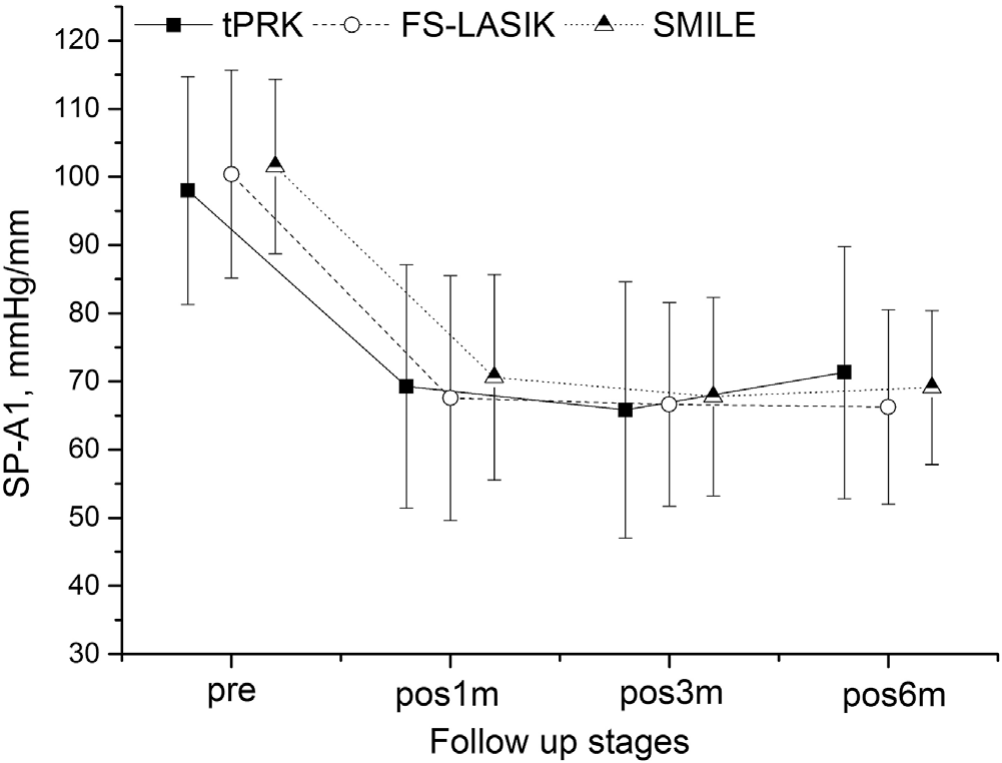
Changes in the stiffness parameter at first applanation (SP-A1) throughout all follow up stages in the transepithelial photorefractive keratectomy (tPRK), femtosecond laser-assisted *in-situ* keratomileusis (FS-LASIK), and small-incision lenticule extraction (SMILE) patient groups (mean and standard deviation).

**FIGURE 2:**
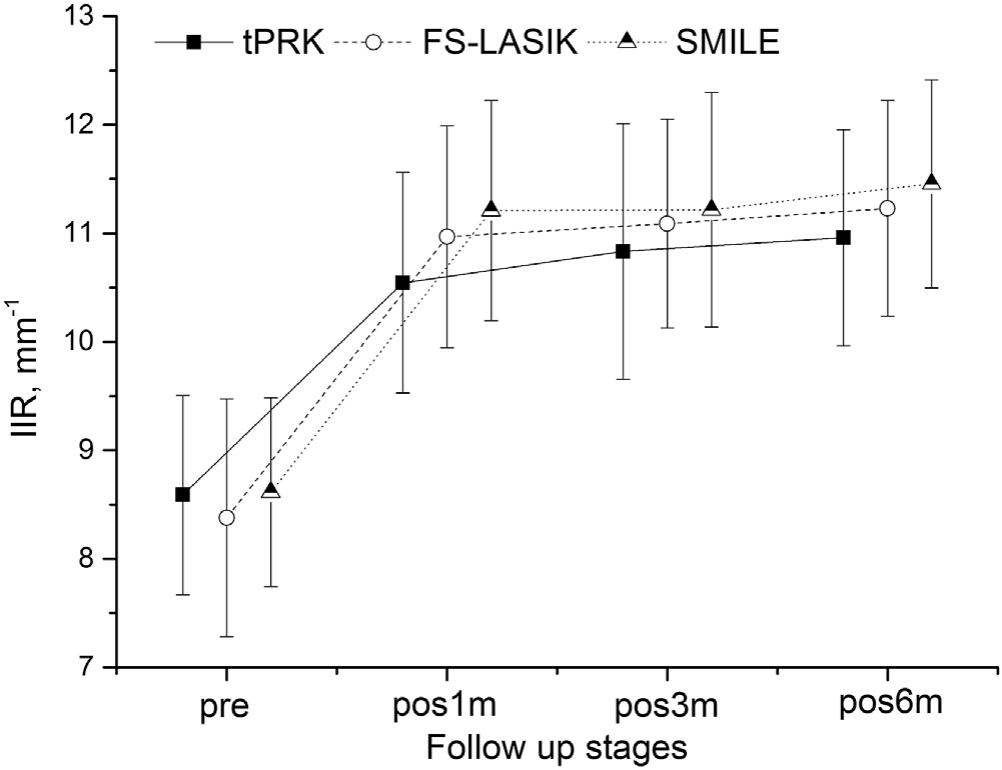
Changes in the integrated inverse radius (IIR) throughout all follow-up stages in the tPRK, FS-LASIK, and SMILE patient groups (mean and standard deviation).

**FIGURE 3:**
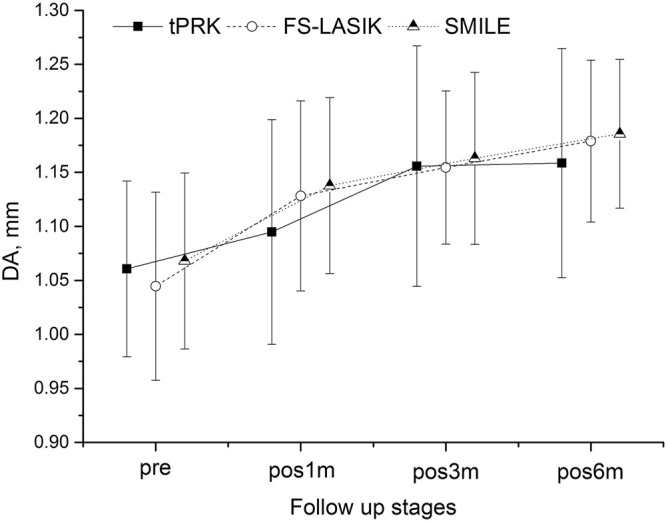
Changes in the deformation amplitude (DA) throughout all follow-up stages in the tPRK, FS-LASIK, and SMILE patient groups (mean and standard deviation).

**FIGURE 4:**
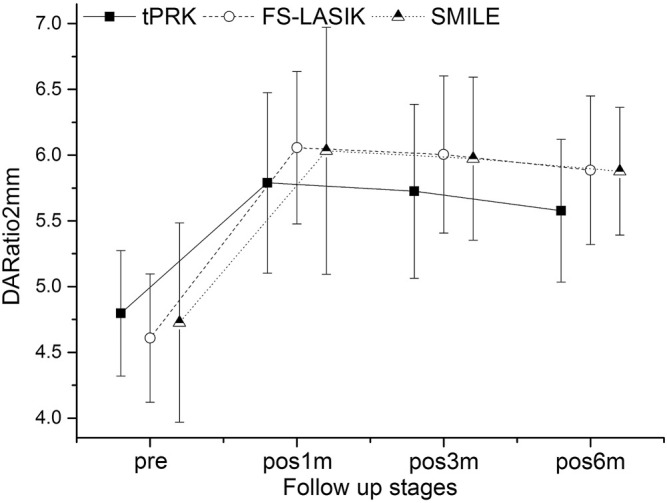
Changes in the deformation amplitude 2 mm away from apex and the apical deformation (DARatio2mm) throughout all follow-up stages in the tPRK, FS-LASIK, and SMILE patient groups (mean and standard deviation).

Figures 1–4 and Table 2 show the corneal biomechanical metrics measured by Corvis ST for the three groups both pre- and post-operation, which represent the effect of the surgical procedure in each patient group. While there were no significant differences (all *p* > 0.05) in SP-A1, IIR, DA, and DARatio2mm among the three surgery groups before surgery, uneven shifts towards overall softening were observed after all three surgery procedures (at pos1m) with small, inconsistent, and insignificant stiffness changes taking place between pos1m and pos6m in most situations.

**TABLE 2:** Biomechanical metrics provided by Corvis ST before and after three forms of corneal refractive surgery.

Biometric parameter	Surgery procedure	Subgroups	Pre	Pos1M	Pos3M	Pos6M	*p* (pre vs pos1m)	*p* (pos1m vs pos6m)
SP-A1, mmHg/mm	tPRK	LM group	96.8 ± 16.6	71.0 ± 16.2	68.5 ± 17.6	74.5 ± 19.4	<0.001	1.000
		HM group	99.2 ± 17.0	67.6 ± 19.4	63.1 ± 19.8	68.0 ± 17.1	<0.001	1.000
		p (LM vs HM)	0.545	0.429	0.218	0.139	—	—
	FS-LASIK	LM group	96.3 ± 12.6	70.2 ± 19.5	70.9 ± 17.0	68.3 ± 13.5	<0.001	1.000
		HM group	103.4 ± 16.4	65.8 ± 16.9	63.9 ± 12.9	64.8 ± 14.8	<0.001	1.000
		p (LM vs HM)	0.031	0.301	0.045	0.274	—	—
	SMILE	LM group	99.1 ± 13.9	69.4 ± 13.9	68.2 ± 14.5	70.0 ± 12.4	<0.001	1.000
		HM group	103.7 ± 11.5	71.7 ± 16.2	67.4 ± 14.9	68.4 ± 10.2	<0.001	0.996
		p (LM vs HM)	0.131	0.522	0.816	0.543	—	—
IIR, mm^−1^	tPRK	LM group	8.69 ± 1.04	10.22 ± 0.98	10.49 ± 1.04	10.64 ± 0.95	<0.001	0.001
		HM group	8.49 ± 0.78	10.86 ± 0.96	11.17 ± 1.22	11.29 ± 0.94	<0.001	0.001
		p (LM vs HM)	0.37	0.006	0.012	0.005	—	—
	FS-LASIK	LM group	8.68 ± 1.08	10.84 ± 1.10	10.97 ± 1.17	11.03 ± 1.00	<0.001	1.000
		HM group	8.16 ± 1.07	11.06 ± 0.97	11.17 ± 0.81	11.38 ± 0.97	<0.001	0.012
		p (LM vs HM)	0.037	0.367	0.389	0.115	—	—
	SMILE	LM group	8.72 ± 0.90	10.87 ± 1.07	10.79 ± 1.00	11.16 ± 1.01	<0.001	0.132
		HM group	8.52 ± 0.85	11.52 ± 0.87	11.6 ± 1.01	11.72 ± 0.83	<0.001	1.000
		p (LM vs HM)	0.313	0.007	0.001	0.012	—	—
DA, mm	tPRK	LM group	1.06 ± 0.09	1.10 ± 0.10	1.15 ± 0.09	1.15 ± 0.11	0.004	0.017
		HM group	1.06 ± 0.07	1.09 ± 0.10	1.17 ± 0.13	1.17 ± 0.10	1.000	<0.001
		p (LM vs HM)	0.765	0.447	0.465	0.491	—	—
	FS-LASIK	LM group	1.04 ± 0.08	1.12 ± 0.09	1.15 ± 0.08	1.16 ± 0.08	<0.001	0.058
		HM group	1.04 ± 0.09	1.13 ± 0.08	1.16 ± 0.06	1.19 ± 0.07	<0.001	<0.001
		p (LM vs HM)	0.979	0.587	0.402	0.074	—	—
	SMILE	LM group	1.06 ± 0.09	1.14 ± 0.09	1.15 ± 0.08	1.18 ± 0.07	<0.001	0.061
		HM group	1.08 ± 0.08	1.14 ± 0.08	1.18 ± 0.07	1.19 ± 0.06	0.001	<0.001
		p (LM vs HM)	0.369	0.797	0.095	0.324	—	—
DARatio2mm	tPRK	LM group	4.85 ± 0.54	5.65 ± 0.51	5.62 ± 0.59	5.44 ± 0.58	<0.001	0.270
		HM group	4.75 ± 0.41	5.93 ± 0.8	5.83 ± 0.72	5.72 ± 0.46	<0.001	0.692
		p (LM vs HM)	0.379	0.087	0.167	0.03	—	—
	FS-LASIK	LM group	4.79 ± 0.45	5.96 ± 0.61	5.89 ± 0.62	5.92 ± 0.64	<0.001	1.000
		HM group	4.48 ± 0.47	6.12 ± 0.56	6.08 ± 0.58	5.86 ± 0.51	<0.001	0.014
		p (LM vs HM)	0.004	0.235	0.191	0.624	—	—
	SMILE	LM group	4.83 ± 1.03	5.95 ± 1.17	5.93 ± 0.55	5.77 ± 0.49	<0.001	0.245
		HM group	4.63 ± 0.37	6.10 ± 0.64	6.01 ± 0.69	5.97 ± 0.46	<0.001	1.000
		p (LM vs HM)	0.276	0.551	0.628	0.075	—	—

SP-A1, stiffness parameter at first applanation; IIR, integrated inverse radius; DA, deformation amplitude, DARatio2mm, deformation amplitude ratio at 2 mm; LM group, low-to-moderate myopia group; HM group, high-myopia group; pre, pos1m, pos3m, and pos6m are the different periods pre- and postoperatively.

SP-A1 decreased at pos1m compared with the pre-surgery stage in all surgery groups (all *p* < 0.01), indicating overall stiffness reduction, then experienced non-significant (*p* > 0.05) fluctuations during follow-up except for the LM-tPRK subgroup (*p* = 0.039, pos3m vs. pos6m). Comparing pos6m and pre, the change in SP-A1 was larger in FS-LASIK (−34.15 ± 13.17 mmHg/mm, significant when compared with tPRK, *p* = 0.008), smaller in SMILE (−32.40 ± 10.42 mmHg/mm, non-significant when compared with tPRK, *p* = 0.090), and smallest in tPRK (−27.40 ± 16.91 mmHg/mm), Figure 1. After correction for CCT_dif_, the changes in SP-A1 between pre and pos6m in tPRK were statistically lower than in FS-LASIK (*p* = 0.001) and SMILE (*p* = 0.022). Furthermore, the decrease in SP-A1 from pre to pos6m in FS-LASIK was statistically higher than in SMILE (*p* = 0.022) after correction for TA. However, after correction for PTA, the changes in SP-A1 between pre and pos6m became non-significant among the three groups (*p* > 0.05). The SP-A1 changes from pre to pos6m were also significantly higher in the HM group than in the LM group after tPRK (*t* = 2.715, *p* = 0.008), FS-LASIK (*t* = 3.876, *p* < 0.001), and SMILE (*t* = 2.626, *p* = 0.011).

IIR exhibited a significant increase from pre to pos1m (*p* < 0.01), demonstrating overall stiffness reduction, and continued to undergo increases, albeit at a much-reduced rate, from pos1m to pos6m in the three groups, Figure 2. The differences between pre and pos6m were statistically significant, being the smallest in tPRK (2.40 ± 0.94 mm^−1^) and relatively higher in SMILE (2.84 ± 1.03 mm^−1^) when compared with tPRK (*p* = 0.020). The IIR changes were also higher in FS-LASIK (2.85 ± 0.96 mm^−1^) compared with tPRK (*p* = 0.014). After correction for CCT_dif_, the changes in IIR between pre and pos6m in tPRK remained lower than in FS-LASIK (*p* = 0.004) and SMILE (*p* = 0.002). Also, after correction for TA, the growth in IIR from pre to pos6m was similar in FS-LASIK and SMILE (*p* = 0.248). However, after correction for PTA, the changes in IIR between pre and pos6m became non-significant among the three groups (*p* > 0.05). The IIR increases from pre to pos6m were further significantly higher in the HM subgroup than in the LM subgroup (tPRK: *t* = −4.678, *p* < 0.001, FS-LASIK: *t* = −4.438, *p* < 0.001, SMILE: *t* = −3.417, *p* = 0.001).

Another evidence of overall stiffness reduction was seen in the significant increases observed in DA at pos1m compared with the pre-surgery stage in all groups (*p* > 0.05 except in the HM subgroup of tPRK). DA then continued to increase in most follow-up stages from pos1m to pos6m. With tPRK, this trend was more obvious from pos1m to pos3m, Figure 3. The change in DA from pre to pos6m in FS-LASIK was significantly higher than that in tPRK (0.134 ± 0.057 mm vs. 0.101 ± 0.086 mm, *p* = 0.009) but was not significantly different from the change in SMILE (0.118 ± 0.063 mm, *p* = 0.409). Meanwhile, the changes in tPRK and SMILE were similar (*p* = 0.448). After correction for CCT_dif_, the changes in DA between pre and pos6m in tPRK remained statistically lower than in FS-LASIK (*p* = 0.024), but not different from SMILE (*p* = 0.750). Meanwhile, the increase in DA from pre to pos6m after FS-LASIK and SMILE was similar (*p* = 0.277) after correction for TA. However, after correction for PTA, the changes in DA between pre and pos6m were larger in FS-LASIK compared with tPRK (*p* = 0.038) but remained similar in SMILE and tPRK (*p* = 1.000). In contrast, the corresponding changes in DA from pre to pos6m were similar in the LM and HM subgroups of tPRK (*t* = −0.855, *p* = 0.396) and SMILE (*t* = 0.084, *p* = 0.934), but not FS-LASIK (*t* = −2.470, *p* = 0.016).

DARatio2mm also significantly increased, denoting overall stiffness reduction, in all three groups from pre to pos1m (all *p* < 0.01). DARatio2mm then experienced a gradual, slight decrease through the rest of the follow-up period in the three groups, Figure 4. The increases in DARatio2mm between pre and pos6m were statistically significant, being the smallest in tPRK (0.79 ± 0.55), higher in SMILE (1.15 ± 0.83) compared with tPRK (*p* < 0.01), and also higher in FS-LASIK (1.28 ± 0.53) when compared with tPRK (*p* < 0.01). After correction for CCT_dif_, the changes in DARatio2mm between pre and pos6m in tPRK were statistically lower than in those in FS-LASIK (*p* = 0.000) and in SMILE (*p* = 0.001). Furthermore, the increase of DARatio2mm from pre to pos6m in FS-LASIK was statistically higher than that in SMILE (*p* = 0.020) after correction for TA. However, after correction for PTA, the changes in DARatio2mm between pre and pos6m were larger in FS-LASIK compared with tPRK (*p* < 0.01) and remained similar in SMILE and tPRK (*p* = 0.416). The DARatio2mm increases from pre to pos6m were further significantly higher in the HM subgroup than in the LM subgroup (tPRK: *t* = −3.309, *p* = 0.001, FS-LASIK: *t* = −2.104, *p* = 0.039; SMILE: *t* = −2.087, *p* = 0.040).

## Discussion

In this study, the biomechanical impact of the three most common LVC procedures was evaluated by monitoring the changes in the *in vivo* biomechanical parameters obtained by the Corvis ST over a 6-month follow-up period. A significant shift in parameter values towards softening was observed following all three surgery forms. After correction for corneal thickness loss (CCT_Diff_) or percentage tissue altered (PTA), the changes in the four biomechanical metrics (SP-A1, IIR, DA, and DARatio2mm), with strong correlation to the cornea’s overall stiffness, showed significant stiffness reductions in all three surgery groups. The metrics’ values also indicated that FS-LASIK was associated with the largest stiffness reduction, followed by SMILE and then tPRK. The results also illustrated continued biomechanical changes during the postoperative period, but these changes were small, inconsistent, and insignificant.

The *in vivo* measurement of corneal biomechanics with air-puff systems like the Corvis ST used in this study was assessed in earlier publications (Miki et al., 2017). Among the several parameters that Corvis ST offers, four were selected for being closely associated with corneal overall stiffness, namely, SP-A1, IIR, DA, and DARatio2mm. In earlier studies, SP-A1 was correlated with removed corneal tissue in refractive surgery procedures (Fernández et al., 2017), DA was influenced by changes in bIOP (Ye et al., 2021), and IIR and DARatio2mm had the highest correlation with CCT (Vinciguerra et al., 2016). All four parameters were also able to detect corneal softening in keratoconus (Roberts et al., 2017; Sedaghat et al., 2018).

In order to minimize the effect of confounding factors that may have an effect on the four biomechanical parameters selected, the groups were paired for age; gender; and baseline CCT, IOP, and biomechanical parameter readings. The additional pairing for surgical parameters was more challenging since different ablation profiles and depths were obtained with different techniques, even with no statistically significant differences being observed in the corrected manifest equivalent and treated optical zones. Moreover, the difference in the planned tissue removal and achieved stromal reduction could be up to 12 µm in SMILE, while in LASIK, it is as small as less than 1 µm (Reinstein et al., 2014; Ryu et al., 2017).

When SMILE was introduced, it was theorized to be the procedure with the least impact on corneal stiffness (Reinstein et al., 2013). Seven et al. (2017) observed less impact on anterior stromal collagen mechanics in SMILE compared to flap-based procedures in numerical modeling. Several clinical studies were conducted to evaluate the difference between the procedures. Guo et al. (2019) carried out a meta-analysis of *in vivo* evaluation of corneal biomechanical properties after the procedures. They observed using the corneal hysteresis (CH) and corneal resistance factor (CRF), from the Ocular Response Analyzer (ORA), that SMILE was superior to FS-LASIK and comparable to PRK. Raevdal et al. (2019), in a systematic review comparing SMILE with flap-based procedures, did not find significant differences between the procedures using CH or CRF. Both systematic reviews included only a limited number of randomized clinical trials and detected a serious risk of bias due to the presence of confounding factors.

Parameters of the relatively new Corvis ST were evaluated in recent studies on refractive surgeries. Cao et al. (2020) studied the effect of FS-LASIK and SMILE using two of the parameters evaluated in this study, DARatio2mm and integrated inverse radius. Although FS-LASIK showed higher post-surgery values of both parameters, indicating higher reductions in stiffness, no significant differences were observed between FS-LASIK and SMILE. The bIOP was also significantly reduced by a similar amount postoperatively at both procedures. Khamar et al. (2019) also reported no significant differences between the procedures in DARatio2mm and integrated inverse radius in a contralateral study with up to 1-month follow-up. They also found no significant differences in SP-A1 or bIOP postoperatively, even though the median value of bIOP was 1.1 mmHg lower in SMILE than in FS-LASIK at the postoperative stage. In another study, Lee et al. (2017a) observed significantly higher increases in DARatio2mm and IIR after FS-LASIK compared to tPRK up to 6 months of follow-up. Meanwhile, there were no significant differences between FS-LASIK and tPRK in the reduction of SP-A1 or bIOP readings.

In this study, with a view to uniformize the data analysis and reduce the confounding factors, the three procedures were evaluated together using data from a single center and a single surgeon. All exams were taken at the same period of the day in order to avoid diurnal variance (Ariza-Gracia et al., 2015). Baseline age, CCT, bIOP, and biomechanical parameters were paired along with surgical parameters including optical zone dimeter and refractive corrections. The main trends reported in the literature that tPRK and FS-LASIK were the procedures with the least and highest effects on corneal biomechanics, respectively, were observed in this study. The SMILE procedure, on the other hand, presented intermediate effects.

Considering the pairwise analysis, the three procedures showed significant shifts towards softening at pos1m in all biomechanical metrics considered (SP-A1, IIR, DA, and DARatio2mm). The increases in DARatio2mm were not significantly different between FS-LASIK and SMILE, but were larger than those recorded after tPRK. This was also observed by Cao et al. (2020) in which the SE and the baseline bIOP values were the closest to the ones in this study, but not by Khamar et al. (2019) or by Lee et al. (2017a), in which the SE was lower and the baseline bIOP values higher than corresponding values in this study.

As a further observation, biomechanical changes induced by the surgical operations were generally larger in the high-myopia group compared to the low-to-moderate myopia group. This was evident in the postoperative changes in SPA1, IIR, DA, and DARatio2mm observed after all three procedures. This outcome is expected as higher degrees of myopic correction typically require more tissue removal and hence can introduce larger reductions in corneal biomechanics. This particular observation was also found in several previous studies (Reinstein et al., 2013; Wang et al., 2014; Fernández et al., 2016; Lee et al., 2017a; Lee et al., 2017b).

By conducting a continuous follow-up, the study also revealed an overall softening trend (although the changes were small and insignificant) in the postoperative stages (pos1m to pos6m) in addition to the immediate effects caused by the surgery (pre to pos1m), similar with the results of an animal test using rabbit done by Raghunathan et al. (2017). The results demonstrated that this trend did not show signs of stopping by the time of pos6m, which was most evident in IIR (Figure 2) and DA (Figure 3). The DARatio2mm however appeared to show an opposite trend with a further decrease from pos1m to pos6m following the immediate increase after surgery at pos1m. At first glance, this finding contradicted the further softening trend suggested by IIR and DA; however, because DARatio2mm is defined as the ratio of deformation amplitude between the corneal apex and the location 2 mm away from it, the results in fact indicated that the corneal deformation amplitude 2 mm away from the apex increased more than that at the apex (DA), which in turn showed non-uniform biomechanical changes (softening) across the cornea. The continuous changes in biomechanical parameters over time and asynchronization of these changes in different regions are believed to be related to the wound healing process after surgery, and they may be the cause of the continuous shape changes after surgery reported above and observed by Bao et al. (2019). The corneal curvature changed statistically significant during the follow-up consisted with that, especially in the FS-LASIK group.

Combined with the correction for corneal thickness loss and percentage tissue altered, which considered the thickness of separated tissue in the FS-LASIK flap and the SMILE cap, the four biomechanical metrics (SP-A1, IIR, DA, and DARatio2mm) showed the largest overall stiffness reductions in FS-LASIK, followed by SMILE, and the lowest stiffness losses in tPRK. These results indicate the negative biomechanical effects of the tissue separation in FS-LASIK flap and SMILE cap, in addition to the tissue ablation in all three procedures. Nevertheless, the decreases in SP-A1 and increases in DARatio2mm from pre to pos6m in FS-LASIK were statistically higher than in SMILE (*p* = 0.022 and 0.020, respectively). These trends illustrate that the SMILE cap was able to contribute to post-surgery corneal stiffness more than an FS-LASIK flap with equal thickness. Separating the tissue flap in LASIK effectively means losing completely this part of the cornea, and along with the tissue ablation, the tissue loss in LASIK is therefore much more than that in tPRK. In SMILE, an attempt is made to maintain some connection between the cap and the rest of the cornea, but this connection is not perfect, as it is affected by the incision and the loss of support on the posterior side. For these reasons, it is expected that tPRK would have a smaller (or much smaller) biomechanical effect on the cornea than both LASIK and SMILE.

The Stress–Strain Index (SSI) was recently introduced as a measure of the material stiffness of corneal tissues (Eliasy et al., 2019). Material stiffness is part of overall stiffness, alongside geometric stiffness, which is not only dominated by corneal thickness but is also influenced by corneal curvature and diameter. With refractive surgery, it is expected that the large thickness loss (due to both tissue ablation and separation) would lead to substantial geometric stiffness loss and significant reductions in corneal overall stiffness. On the other hand, the material stiffness would not be expected to undergo notable changes, as these would be limited to the small effects of wound healing following surgery (Dupps and Wilson, 2006). For these reasons, the SSI (measure of material stiffness) would not be expected to demonstrate significant changes, unlike those taking place in the overall stiffness parameters like the SP and IIR. Presenting these small changes in SSI alongside the large changes in the overall stiffness metrics could therefore be confusing—and in any case not relevant to the subject of this study—and for this reason, SSI was not included in the present comparisons.

The study included a number of limitations, one of which was the diurnal fluctuations of the IOP that were reported to influence the values of the four Corvis ST metrics considered (Ye et al., 2021). The variations observed between the pre- and post-surgery stages could have been affected by this change. Furthermore, the use of different femtosecond lasers in FS-LASIK and SMILE, which was necessary due to the Wenzhou Eye Hospital surgical routine, may have led to different flap and cap architectures, although the differences were not significant (Riau et al., 2014).

In conclusion, the biomechanical impact of tPRK, FS-LASIK, and SMILE varied significantly in this study where the data was paired for the main confounding factors. The SMILE procedure induced less corneal biomechanical degradation than FS-LASIK but more than tPRK in cases with comparable corrected refractive errors and optical zone diameter.

## Data Availability

The raw data supporting the conclusion of this article will be made available by the authors, without undue reservation.
